# Optical coherence tomography otoscope for imaging of tympanic membrane and middle ear pathology

**DOI:** 10.1117/1.JBO.29.8.086005

**Published:** 2024-08-20

**Authors:** Wihan Kim, Ryan Long, Zihan Yang, John S. Oghalai, Brian E. Applegate

**Affiliations:** a University of Southern California, Caruso Department of Otolaryngology–Head & Neck Surgery, Los Angeles, California, United States; b University of Southern California, Alfred Mann Department of Biomedical Engineering, Los Angeles, California, United States

**Keywords:** optical coherence tomography, hearing, ear

## Abstract

**Significance:**

Pathologies within the tympanic membrane (TM) and middle ear (ME) can lead to hearing loss. Imaging tools available in the hearing clinic for diagnosis and management are limited to visual inspection using the classic otoscope. The otoscopic view is limited to the surface of the TM, especially in diseased ears where the TM is opaque. An integrated optical coherence tomography (OCT) otoscope can provide images of the interior of the TM and ME space as well as an otoscope image. This enables the clinicians to correlate the standard otoscopic view with OCT and then use the new information to improve the diagnostic accuracy and management.

**Aim:**

We aim to develop an OCT otoscope that can easily be used in the hearing clinic and demonstrate the system in the hearing clinic, identifying relevant image features of various pathologies not apparent in the standard otoscopic view.

**Approach:**

We developed a portable OCT otoscope device featuring an improved field of view and form-factor that can be operated solely by the clinician using an integrated foot pedal to control image acquisition. The device was used to image patients at a hearing clinic.

**Results:**

The field of view of the imaging system was improved to a 7.4 mm diameter, with lateral and axial resolutions of 38  μm and 33.4  μm, respectively. We developed algorithms to resample the images in Cartesian coordinates after collection in spherical polar coordinates and correct the image aberration. We imaged over 100 patients in the hearing clinic at USC Keck Hospital. Here, we identify some of the pathological features evident in the OCT images and highlight cases in which the OCT image provided clinically relevant information that was not available from traditional otoscopic imaging.

**Conclusions:**

The developed OCT otoscope can readily fit into the hearing clinic workflow and provide new relevant information for diagnosing and managing TM and ME disease.

## 1 Introduction

The way in which physicians view the tympanic membrane (TM) and middle ear in the clinic has not changed much over the last 150 years. A speculum is placed into the ear canal with lens-aided viewing and illumination through the aperture of the speculum. The most common speculum used today was invented by A. Hartmann in Berlin in 1881.^1^ Since then, illumination and optics have improved but little else has changed other than the addition of a digital camera for video otoscopy. Routine otoscopic imaging yields information on the surface of the TM, but with no depth perception because only a monocular view can be obtained. If the membrane is transparent, the ossicles just below may be discerned and used as a reference for TM depth. However, no quantitative three-dimensional image is possible, and in many pathological cases the TM is opaque, preventing visualization of the ossicular chain. Subjective interpretation varies with physician experience^2^^,^^3^ and can lead to incorrect or delayed diagnosis. For example, the United States pediatrician rate of successful otitis media diagnosis is 51% with a false positive rate of 26% as the diagnosis is mostly based on clinical presentation.^4^ Modern medical imaging approaches such as MRI and CT are only sparingly utilized because they are expensive, require a second visit by the patient, and only yield relatively poorly resolved images of the tympanic membrane and middle ear.

Pathologies of the middle ear are composed of a heterogeneous group of diseases that pose a significant risk of hearing loss, facial nerve paralysis, and other serious complications due to their impact on important middle ear and surrounding structures.^5^ Some of the more common diseases include otitis media and cholesteatomas, with an estimated 80% of children by age 3 having one or more episodes of otitis media and an annual incidence of acquired cholesteatomas being 9 to 12.6 per 100,000 people.^6^^,^^7^ Less common pathologies include more insidious neoplastic middle ear lesions (e.g., facial neuromas, glomus tumors).^8^^,^^9^ Together, they are major contributors to the over 430 million people globally to have acquired disabling hearing impairment (>35 to 40 dBA for adult and >30  dBA for children unaided hearing threshold level).^10^^,^^11^ The accurate diagnosis of these diseases requires clinicians to pay close attention to subtle changes in normal physiology that are sometimes missed or cannot be detected with existing diagnostic imaging without direct surgical confirmation.^12^^,^^13^

Optical coherence tomography (OCT), an interferometry-based non-invasive imaging modality that provides 3D morphological images by capturing the back-scattered light as a laser scan across the biological tissues of interest, is currently being explored as a potential tool to fill the gap in anticipatory diagnostic imaging of the ear by providing structural and functional information not available from traditional diagnostic methods.^14^ It has already been used to help better characterize and diagnose various pathologies of the middle ear and TM.^15^ It has been used to diagnose chronic and acute otitis media with effusion with greater accuracy than traditional otoscopes,^16^ intraoperatively characterize sclerotic changes in patients with otosclerosis,^17^ and differentiate between cholesteatoma and inflammatory tissue.^18^ OCT has also been used to characterize the severity of pathological changes in the TM from diseases that affect the TM such as myringitis.^19^ In addition, compared with traditional imaging modalities, such as MRI and CT, OCT is less expensive, more portable, does not involve ionizing radiation, and has better resolution, traits that make it advantageous as a diagnostic imaging tool for middle ear pathologies in a busy clinical setting.^20^^,^^21^

We have designed a hand-held OCT otoscope with a wide field of view that is capable of imaging nearly the entire tympanic membrane and middle ear down to the cochlear promontory.^22^ The integrated fiber-optic interferometer has a similar performance to our prior free-space approach with demonstrated sub-nanometer sensitivity to vibration.^23^ This system, with high performance and a small form factor, is well suited for use in the clinic. In this study, we demonstrate the clinical utility of our device to characterize a variety of middle ear pathologies in a large population of clinic patients. In addition, we present cases in which our device was able to provide information not able to be obtained using traditional imaging techniques. The findings of this study further support the merit of OCT as a clinical diagnostic tool that provides clinicians with information to aid in the early detection and management of various middle ear pathologies.

## 2 Method

### 2.1 OCT Otoscope System

**Fig. 1:**
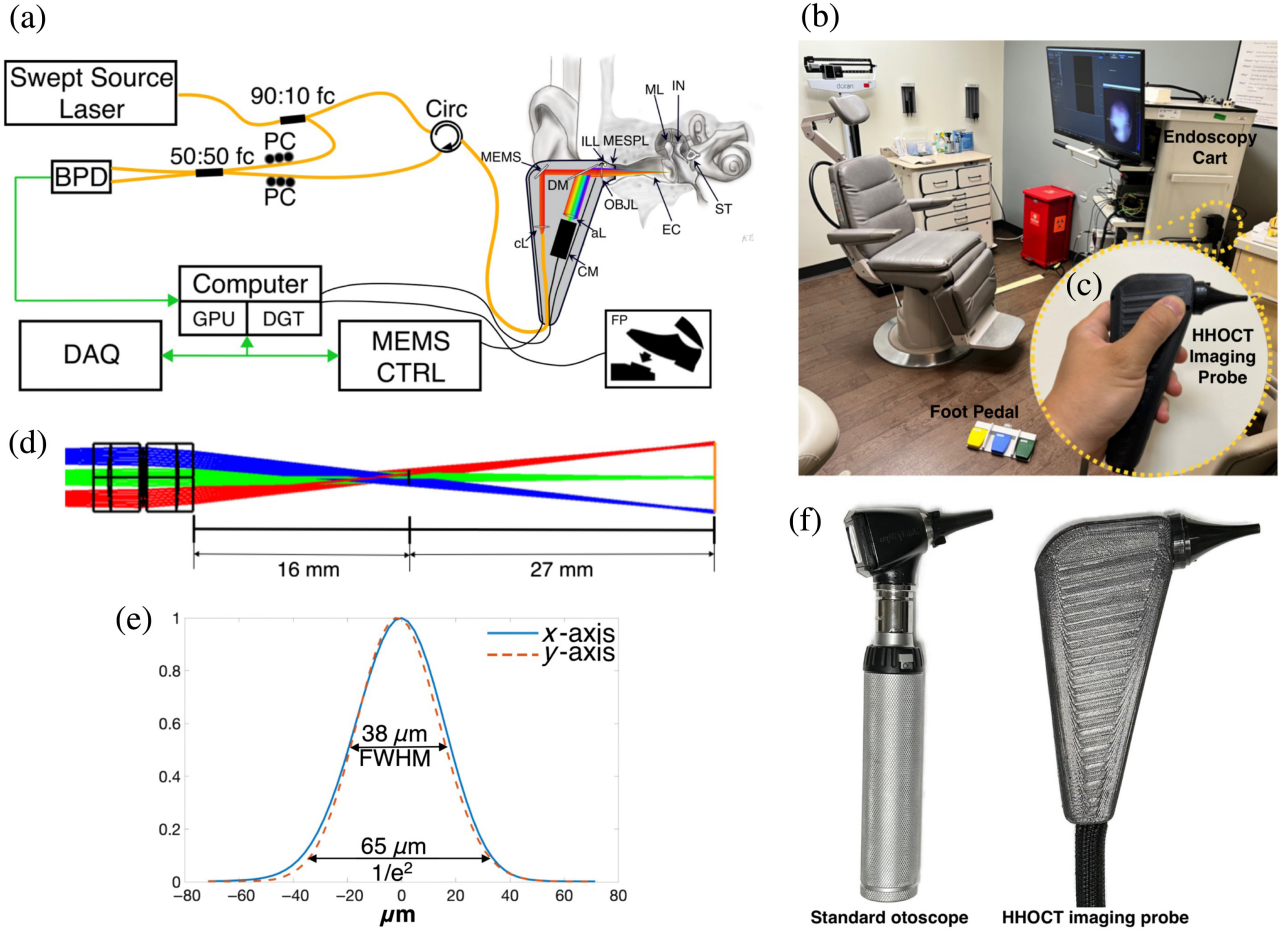
System configuration (a) schematic diagram. Abbreviations: 90:10 fc, 90:10 fiber fused coupler; Circ, fiber optic circulator; cL, collimating lens; MEMS, MEMS scan mirror; DM, dichroic mirror; ILL, illuminator; OBJL, objective lens; MESPL, middle ear speculum; EC, ear canal; ML, malleus; IN, incus; ST, stapes; aL, achromatic lens; CM, video camera; PC, polarization controller; 50:50 fc, 50:50 fiber fused coupler; BPD, balanced photo-detector; GPU, graphic processing unit; DGT, digitizer; DAQ, data acquisition board, MEMS CTRL, MEMS scan mirror controller; FP, foot pedal. (b) Entire system setup in a regular clinic, (c) close-up of the hand-held imaging probe. (d) Ray tracing diagram in Zemax. (e) Lateral point-spread function (PSF) of the x and y axis measured at the focal plane and (f) size comparison of a standard otoscope and the HHOCT imaging probe.

Conceptually, the hand-held OCT (HHOCT) clinical system design (see Fig. 1) follows our previous study that reported the otoscopic OCT system.^22^ It is comprised of several components: the hand-held imaging probe, an endoscopy cart, and a wireless foot pedal serving as an input assist device. The system employed a swept laser with a center wavelength of 1310 nm and a 39 nm bandwidth, yielding an axial resolution of 33.4  μm (in tissue, n=1.3) for OCT imaging, which is sufficient for resolving the human middle ear structures.^24^

The essential optical system design is shown in Fig. 1(a). The swept laser output was directed into a 90:10 optical fiber coupler (fC). The 10% side illuminated the reference arm of the interferometer. After passing through a fiber optic patch cable sized to an optical path length matched with the sample arm, the reference light passed into a 50:50 fC where it was combined with the sample arm. The 90% side illuminated the sample arm, passing through a circulator (Circ) and then onto the hand-held imaging probe. Inside the hand-held imaging probe, the light was guided by a dual axes MEMS mirror (Mirrocle Technology, Northamptonshire, United Kingdom) through the objective lens and onto the sample for scanning. The back-reflected light passed back through the circulator and was combined with the reference arm via the 50:50 optical fC. The two outputs of the fC were directed into the balanced photodetector (BPD, WL-BPD600MA, Wieserlabs, Penzberg, Germany).

The objective yielded a 38  μm (FWHM) scanning beam spot at the focal plane measured by a commercial scanning-slit optical beam profiler (BP104-IR, Thorlabs, Newton, New Jersey, United States) and 5.03 mm depth of focus (DOF) calculated from the waist ($1/e^{2}$) of a Gaussian beam, as shown in Fig. 1(e). A ray tracing diagram for the objective lens is shown in Fig. 1(d). As can be seen, the rays cross at ∼16  mm from the final lens surface at a point coinciding approximately with the tip of the speculum. From that point, the rays fan out and come to a focus at an arc about 27 mm from the crossing. As a result, the image is sampled in a spherical polar coordinate system, unlike our prior telecentric system, which naturally samples in the more convenient Cartesian space. Below, we describe our approach to resampling in Cartesian space and aberration correction. The advantage of this imaging pattern is a larger field of view, 7.4 mm at the focal plane through the limited aperture of the middle ear speculum. This represents an approximately two-fold increase over our prior telecentric system.

A high-pass dichroic mirror (DM) positioned between the MEMS mirror and an achromatic objective lens (aL) directed the back-reflected visible light onto a small CMOS sensor (MU9PC-MH, XIMEA, Münster, Germany) within the hand-held imaging probe to generate a video otoscope image. Visible light illumination of the ear canal was accomplished by a set of visible LEDs mounted around the objective lens. Real-time video output from the CMOS was shown on the monitor and used by the clinician to guide the hand-held unit to the proper position for OCT imaging. As a size comparison of our HHOCT imaging probe with a typical otoscope, we photographed the two side-by-side as shown in Fig. 1(f). The outer shell of the HHOCT device is designed to resemble the grip angle and size of a standard otoscope. This design choice aims to make it familiar for the clinician and ensure patient comfort during examinations.

The endoscopy cart housed the major electronic components. Detailed information about the electronic devices and signal processing procedures can be found in previous studies.^22^^,^^23^ Briefly, the interferometric signal from the BPD was connected to a digitizer and processed using custom software written in Python, C++, and Cuda C. The 400 MHz clock from the swept laser served as the master clock. The data acquisition (DAQ) board generated an enable trigger that worked as a logical AND so that the digitizer only acquired data during the desired portion of the mirror scan. The DAQ also generated a synchronized analog signal for controlling the MEMS mirror. In addition, the foot pedal facilitated hands-free data acquisition, minimizing motion artifacts caused by the operator. The foot pedal, operated through the Bluetooth Secure Personal Network (PAN), was paired with the system software for image capture that allowed a single operator hands-free operation for the standalone and meticulous measurements in clinical procedures.

As noted above, the new imaging device has a much larger field of view than our prior telecentric systems. As a consequence, we sample in spherical polar space rather than the typical Cartesian space as well as introduce some image aberration. To correct this and enable accurate image dimensions, we developed a two-part algorithm that includes coordinate transformation and aberration correction.

### 2.2 Model for Coordinate Transformation

**Fig. 2:**
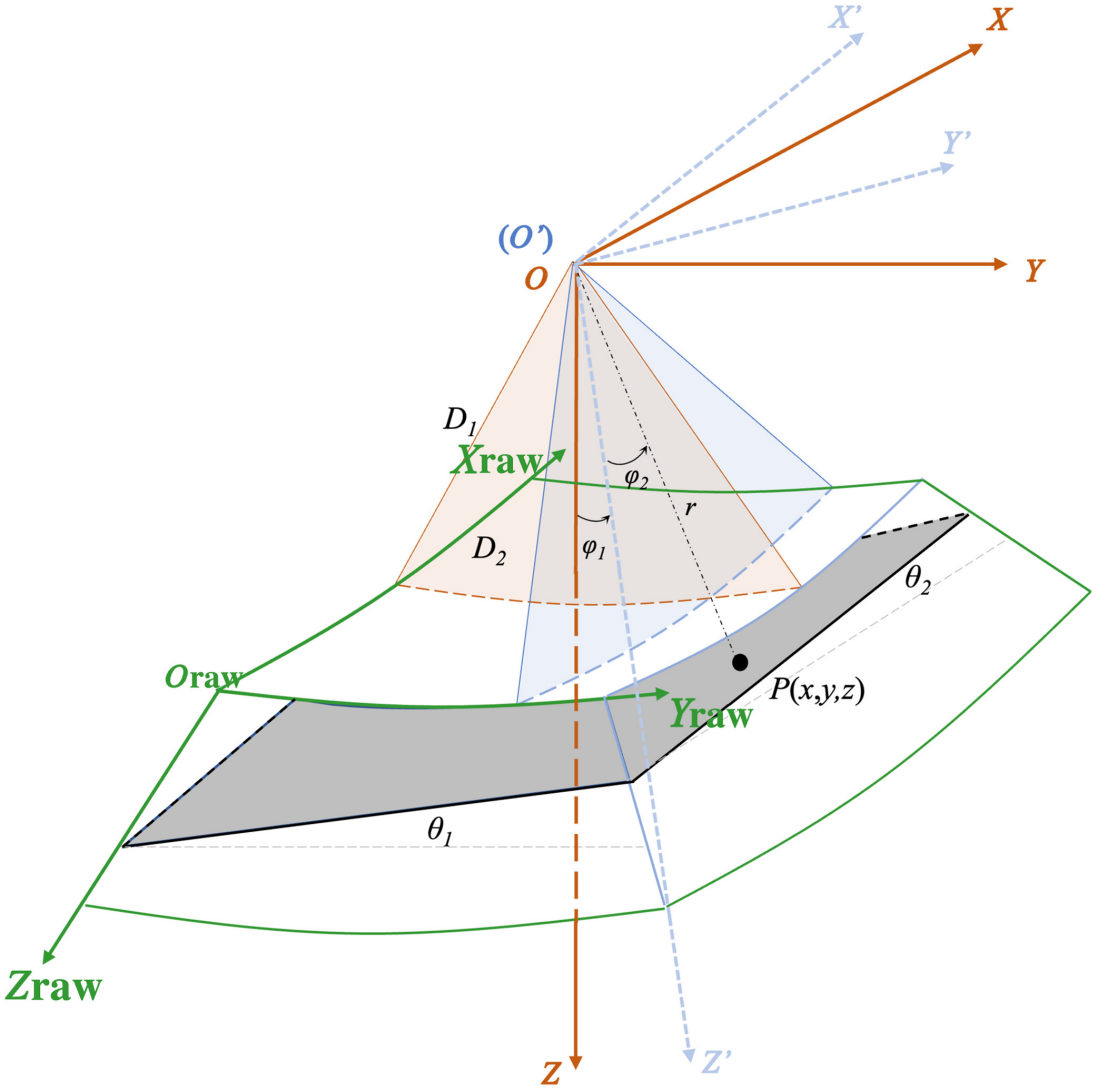
Model of the coordinate transformation for the otoscopic OCT system. The green 3D shape represents the collected data. The fast-axis and slow-axis coordinate systems are shown in blue and red, respectively. The gray plane represents an arbitrary plane represented in both spaces.

Although our OCT system nominally scans in spherical polar coordinates, we chose to treat it as two polar coordinate systems, in part because each axis of the MEMs mirror has its own hysteresis and center of rotation. Hence, OCT volume scanning involves two steps: x-z plane scanning (fast axis) and y-z plane scanning (slow axis). As shown in Fig. 2, we establish two Cartesian coordinate systems to represent the real sample space; one follows the slow axis $R_{C1}(O,X,Y,Z)$, and the other is based on the fast axis $R_{C2}(O^{\prime },X^{\prime },Y^{\prime },Z^{\prime })$, with $O(O^{\prime })$ representing the mirror rotation center. A polar coordinate system $R_{\text{raw}}(O_{\text{raw}},x_{\text{raw}},y_{\text{raw}},z_{\text{raw}})$ is introduced to represent the measured image space.

Assuming a point P in the plane is denoted as $P_{1}(x_{1},y_{1},z_{1})$ in $R_{C1}$ and $P_{2}(x_{2},y_{2},z_{2})$ in $R_{C2}$, we establish the relationship between them as

$$P_{1}=M*P_{2},$$ (1)

where M represents the transformation matrix,

$$M=[\begin{array}{cccc} 1 & 0 & 0 & 0\\ 0 & \cos(\varphi_{1}) & \sin(\varphi_{1}) & \left(D_{1}-D_{2})\sin(\varphi_{1}\right)\\ 0 & -\sin(\varphi_{1}) & \cos(\varphi_{1}) & \left(D_{1}-D_{2})\cos(\varphi_{1}\right)\\ 0 & 0 & 0 & 1 \end{array}],$$ (2)

where $\varphi_{1}$ is the angle between two axes as defined in Fig. 2.

According to Eqs. (1) and (2), we derive

$$\left\{ \begin{array}{l} x_{1}=x_{2}\\ y_{1}=\cos(\varphi_{1})y_{2}+\sin(\varphi_{1})z_{2}+(D_{1}-D_{2})\sin(\varphi_{1})\\ z_{1}=-\sin(\varphi_{1})y_{2}+\cos(\varphi_{1})z_{2}+(D_{1}-D_{2})\cos(\varphi_{1}) \end{array}.\right.$$ (3)

Furthermore, we represent the equation of the plane in $R_{C1}$ as

$$z_{1}=x_{1}\;\tan(\theta_{1})+y_{1}\;\tan(\theta_{2})+(d+D_{1}).$$ (4)

The point P is represented in $R_{C2}$ as

$$x_{2}=r_{2}\;\sin(\varphi_{2}),$$ (5)

$$z_{2}=r_{2}\;\cos(\varphi_{2}),$$ (6)

$$\varphi_{2}=\tan^{-1}(x_{2},z_{2}),$$ (7)

$$r_{2}=\sqrt{x_{2}^{2}+z_{2}^{2}}.$$ (8)

Therefore, we derive the following equation:

$$r_{2}(\cos(\varphi_{1})\cos(\varphi_{2})-\sin(\varphi_{2})\tan(\theta_{1})-\sin(\varphi_{1})\cos(\varphi_{2})\tan(\theta_{2}))\;=d+D_{1}+(D_{1}-D_{2})(\sin(\varphi_{1})\tan(\theta_{2})-\cos(\varphi_{1})).$$ (9)

In the polar space, we know point P is represented as

$$x_{\text{raw}}=x_{0}+\varphi_{2}D_{2},$$ (10)

$$y_{\text{raw}}=y_{0}+\varphi_{1}D_{1},$$ (11)

$$z_{\text{raw}}=r_{2}-D_{2}.$$ (12)

Hence, $\varphi_{1}$ and $\varphi_{2}$ are given as

$$\varphi_{1}=(x_{\text{raw}}-x_{0})/D_{2},$$ (13)

$$\varphi_{2}=(y_{\text{raw}}-y_{0})/D_{1}.$$ (14)

Finally, we define the expression of each axial profile position $z_{\text{raw}}$ in $R_{\text{raw}}$ as

$$z_{\text{raw}}=\frac{d+D_{1}+(D_{1}-D_{2})[\sin(\frac{y_{\text{raw}}-y_{0}}{D_{1}})\tan(\theta_{2})-\cos(\frac{y_{\text{raw}}-y_{0}}{D_{1}})]}{\cos(\frac{y_{\text{raw}}-y_{0}}{D_{1}})\cos(\frac{x_{\mathrm{raw}}-x_{0}}{D_{2}})-\sin(\frac{x_{\text{raw}}-x_{0}}{D_{2}})\tan(\theta_{1})-\sin(\frac{y_{\mathrm{raw}}-y_{0}}{D_{1}})\cos(\frac{x_{\text{raw}}-x_{0}}{D_{2}})\tan(\theta_{2})}-D_{2}.$$ (15)

We initially constructed the model with six parameters ($\left(x_{0},y_{0},D_{1},D_{2},\theta_{1},\theta_{2}\right)$) based on Eq. (15). The root-mean-square error (RMSE) was used as the performance indicator for the optimization; it is given as

RMSE=1mn∑1m∑1n(zraw(i,j)−z^(i,j))2, (16)

where m and n represent the axial and lateral pixel numbers in the OCT images, respectively, and z^ denotes the values obtained from the model.

### 2.3 Model for Aberration Correction

**Fig. 3:**
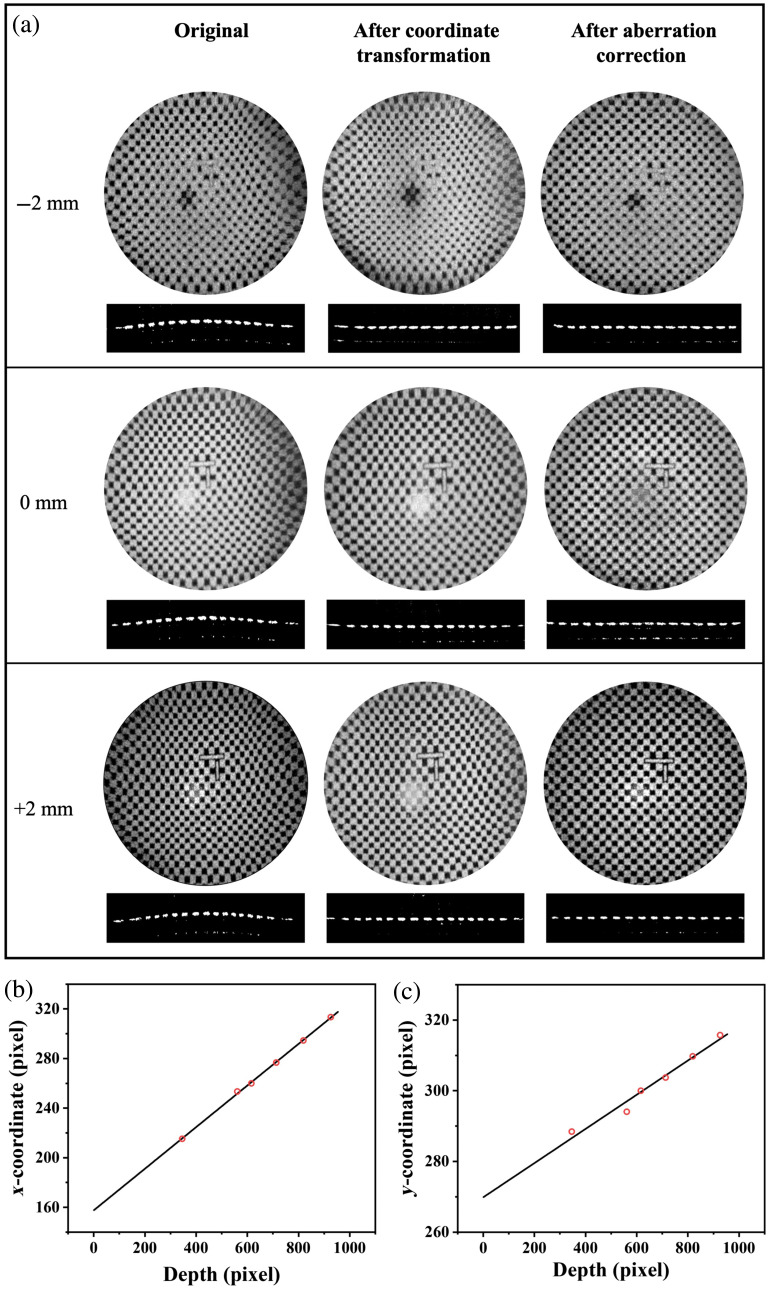
(a) Calibration target images at the focal plane (0 mm) and ±2  mm. Enface images at the depth of the target, above a B-scan through the middle of the target. (b) and (c) Representative linear fits of corresponding x and y pixel positions as a function of depth.

After performing a coordinate transformation, the OCT image exhibited distortion attributed to optical aberrations, as illustrated in Fig. 3(a). We imaged a checkerboard calibration target (Edmund Optics Inc., Barrington, New Jersey, United States), 0.2×0.2  mm square pattern, at various imaging depths while controlling the spatial offset to assess the image quality and the extent of the aberrations. We estimated the residual aberration present in depth slices of an OCT volume by constructing a polynomial model based on the coordinate correspondence between the OCT enface images with the standard checkerboard pattern image serving as the reference.

### 2.4 Clinical Imaging Protocol

This study was approved by the institutional review board at the University of Southern California (HS-21-00338). Over 100 patients were imaged after providing informed consent in a tertiary care otology clinic at Keck Hospital of USC from October 2022 to June 2023. Patients’ ears were cleaned of wax with a curette prior to imaging. Inclusion criteria included anyone with a patent ear canal with visibility of the TM on the otoscopic view. Exclusion criteria included patients with active bleeding, discharge, or open wound in the external ear canal; a non-visible TM; or a non-patent ear canal. Patients were imaged while sitting upright in an exam chair. Both ears were imaged unless a patient had abnormal anatomy or intolerance to the plastic ear speculum that made imaging one ear not possible within a clinic setting.

Multiple OCT volumes were obtained from each of the four anatomical quadrants of the TM using superficially identified anatomical landmarks (e.g., Umbo, pars flaccida) on live otoscopic video feed to orient the scanning volume location and corroborate ME structures seen in real time OCT B scans. A single volume scan took 0.4 s. The relatively high speed helped to reduce motion artifacts that may arise from patient or operator movement. The region of interest was selected with the center of the OCT beam positioned directly over the umbo. This was done to encompass the entire tympanic membrane (TM) and cover the area with suspected pathology, thereby maximizing the likelihood of capturing intriguing pathological features within the OCT volumes. The total imaging time, including consenting the patient, reviewing the images, and explaining to patients what they were seeing, was <5  min. OCT volumes were post-processed and analyzed using MatLab (MathWorks), Amira (Thermofisher Scientific), and Fiji (NIH).

## 3 Results

### 3.1 Coordinate Transformation and Aberration Correction

We found the following parameters in Table 1 for mapping our volumetric images into Cartesian space. Examples of images at the focal plane and ±2  mm before and after coordinate transformation are shown in Fig. 3(a) columns 1 and 2. The imaging target has 200  μm squares with an asymmetric cross near the center of the image to aid in registration. As can be seen, although the image quality is improved, there is still aberration, resembling barrel distortion, that can be corrected.

**Table 1:** Parameters of coordinate transformation.

$x_{0}$ (pix)	$y_{0}$ (pix)	$D_{1}$ (pix)	$D_{2}$ (pix)	$\theta_{1}$ (deg)	$\theta_{2}$ (deg)
166.3	60.08	1432	1479	0.002288	−0.07968

We performed aberration correction using the same checkerboard calibration target at six different depths ranging from −5 to 5 mm. Between these depths, we assumed a linear relationship. As demonstrated in Figs. 3(b) and 3(c), the linear fit among corresponding points has an $R^{2}$ of 0.99 and 0.97, respectively. Therefore, we can readily realize the aberration correction of 3D OCT images by linear fitting. The result following this algorithm is the third column in Fig. 3(a), i.e., after coordinate transformation and aberration correction.

### 3.2 Normal TM and Middle Ear

**Fig. 4:**
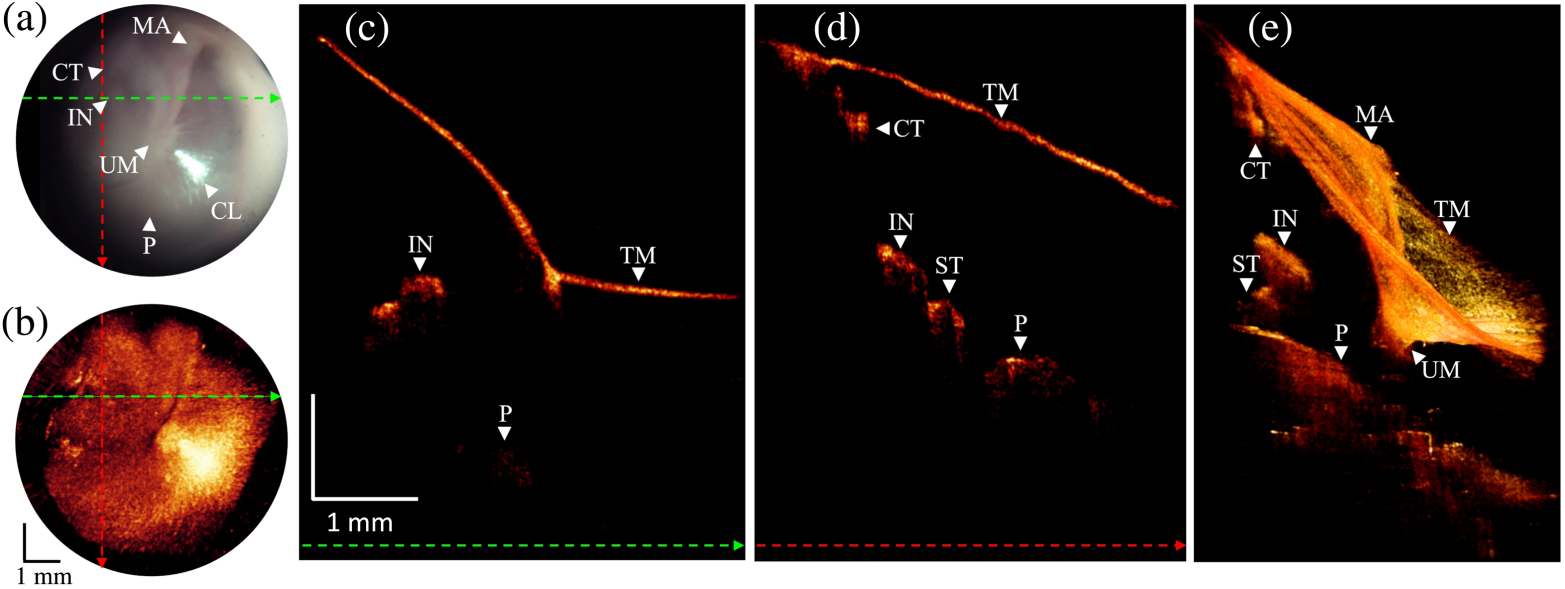
Otoscope and OCT image acquired from normal ear. Abbreviations: MA, malleus; CT, chorda tympani; TM, tympanic membrane; IN, incus; UM, umbo; CL, cone of light; P, promontory. (a) A snapshot of the otoscopic video camera. Dashed colored lines indicate the positions where the linear B-scans are shown in panels (c) and (d). (b) *En face* image generated by SVP from an OCT volume. Dashed colored lines indicate the positions where the linear B-scans are shown in panels (c) and (d). (c) B-scan extracted along the green-colored dashed line from the OCT volume. (d) B-scan extracted along the red-colored dashed line from the OCT volume. (e) 3D volume rendering.

Here, we present several example cases from the clinical study. Our first example is a patient with a healthy middle ear as confirmed by an expert otologist through clinical assessment and otoscopic examination. From the otoscopic view in Fig. 4(a), the tympanic membrane can be seen to be fairly transparent, which allows us to see the incus, malleus, and cochlear promontory through the membrane. Other anatomical landmarks include the umbo and cone-of-light. Figure 4(b) is a summed voxel projection (SVP) of the OCT volume in which the 2D image is generated by summing along the depth dimension. This generates an image that can be directly compared with the standard otoscope view to verify orientation. Figures 4(c) and 4(d) are representative cross-sectional B-scans that were extracted along the dashed colored lines, as shown in panels (a) and (b). The cross-sections show a normal thin tympanic membrane along with sections of the chorda tympani and stapes in addition to the anatomy that was also visible in the otoscopic view. Figure 4(e) displays the 3D volume rendering that visualizes major anatomical structures presented in the otoscopic view.

### 3.3 Abnormal Tissue Growth

**Fig. 5:**
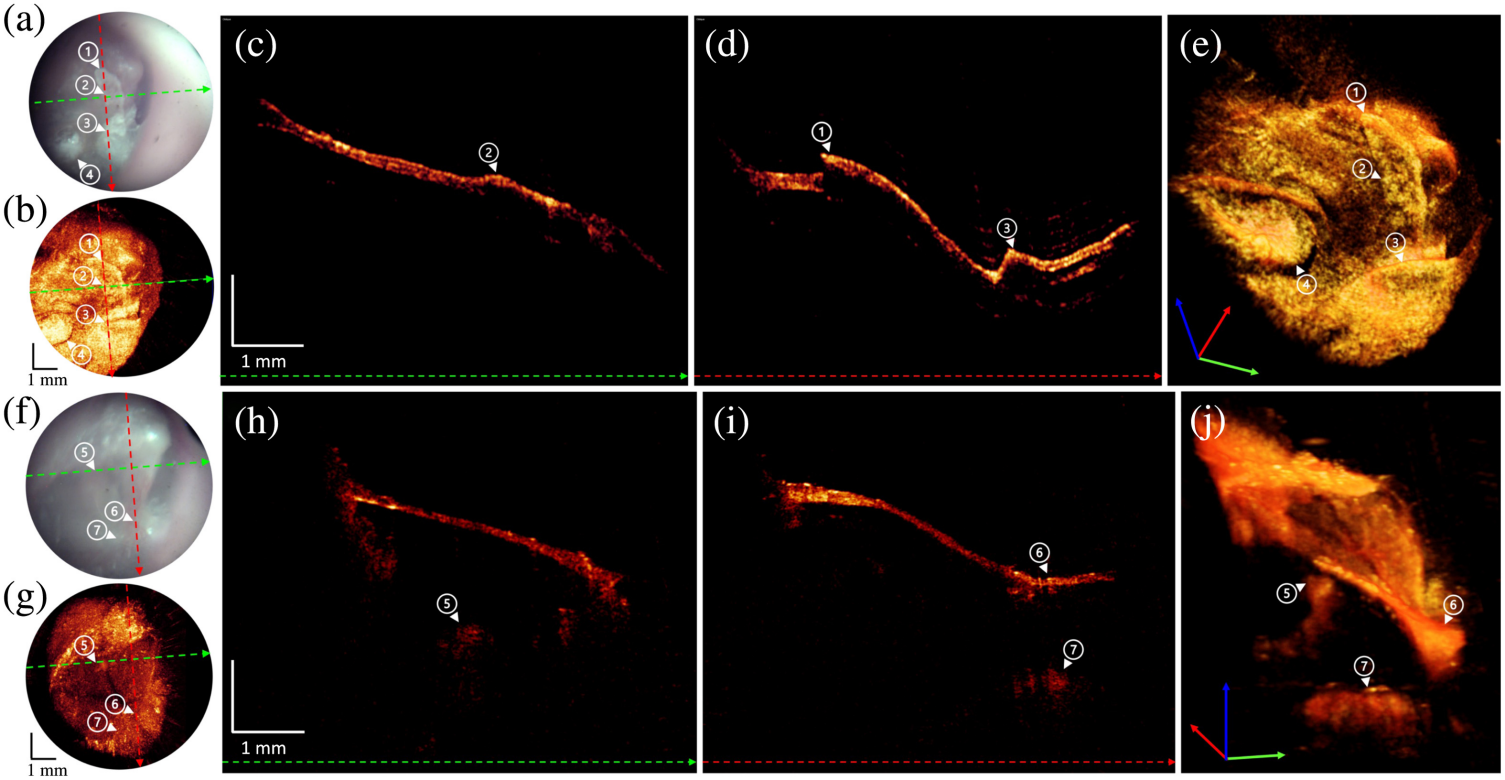
Otoscope and OCT image acquired from myringitis over the subsequent visits. (a) A snapshot of the otoscopic video camera at first visit. Dashed colored lines indicate the positions where the linear B-scans are shown in panels (c) and (d). (b) *En face* image generated by SVP from an OCT volume at first visit. Dashed colored lines indicate the positions where the linear B-scans are shown in panels (c) and (d). (c) B-scan extracted along the green-colored dashed line from the OCT volume at first visit. (d) B-scan extracted along the red-colored dashed line from the OCT volume. White arrows with circled numbers from 1 to 4 denote the layered plaques of abnormal tissue partially adhere to the TM. (e) Frame from (Video 1) showing 3D volume rendering and B-scan fly through at first visit. (f) A snapshot of the otoscopic video camera at the visit 28 days after the first visit. Dashed colored lines indicate the positions where the linear B-scans are shown in panels (h) and (i). (g) *En face* image generated by SVP from an OCT volume at the visit 28 days after the first visit. Dashed colored lines indicate the positions where the linear B-scans are shown in panels (h) and (i). (h) B-scan extracted along the green-colored dashed line from the OCT volume at the visit 28 days after the first visit. (i) B-scan extracted along the red-colored dashed line from the OCT volume. White arrows with circled numbers from 5 to 7 denote the symbolic middle ear structures. The circled number 5 indicates the incus. The circled number 6 shows the umbo. The circled number 7 is from a part of a cochlear promontory. (j) Frame from (Video 2) showing 3D volume rendering and B-scan fly through at 28 days after the first visit. (Video 1, MP4, 2.50 MB [URL: https://doi.org/10.1117/1.JBO.29.8.086005.s1]; Video 2, MP4, 2.36 MB [URL: https://doi.org/10.1117/1.JBO.29.8.086005.s2]).

This case is a patient presented with 2 weeks of ear pain and aural fullness. An otoscopic evaluation by an expert otologist revealed what appeared to be abnormal tissue growth superficially on bilateral TMs. The level of involvement with the TM and if the tissue was directly against the TM were not discernible with a routine physical exam. The physician used OCT imaging to determine if tissue debridement was possible in the clinic without damage to the TM. Representative images are shown in Fig. 5.

Panels (a) and (b) are the otoscopic image and SVP. Panels (c) and (d) are representative cross-sectional OCT B-scans that show the layered plaques of abnormal tissue partially adherent to the surface of the TM with partial elevation of the TM. The plaques are strongly scattering and therefore strongly shadow the tissue below. This leads to the sharp edges labeled (1) and (2) in panel (d) that look like discontinuities in the TM. Panel (e) displays a still image from a movie showing the 3D volume rendering. The volume rendering helps visualize the plaques.

These are denoted with white arrows and circled numbers. Given the findings, a conservative management approach with Debrox drops (Prestige Consumer Healthcare Inc., Irvington, New York, United States) was chosen. The patient was imaged every week over four subsequent visits. Images from the final visit are shown in Figs. 5(f)–5(j). The images clearly show the resolution and clearance of abnormal tissue. The images revealed no residual tissue plaques, a thin normal-looking TM in cross-section, and middle ear anatomy such as the incus and cochlear promontory. Panel (j) displays a still image from a movie showing the 3D volume rendering. The volume rendering helps show how the plaques have largely been resolved.

### 3.4 Chronic Otitis Media with Effusion

**Fig. 6:**
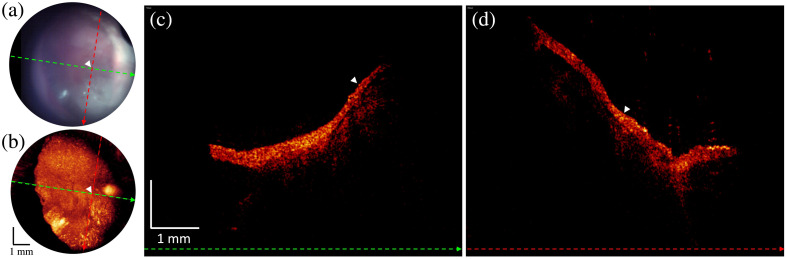
Otoscope and OCT image acquired from effusion. (a) A snapshot of the otoscopic video camera at first visit. Dashed colored lines indicate the positions where the linear B-scans are shown in panels (c) and (d). (b) *En face* image generated by SVP from an OCT volume at first visit. Dashed colored lines indicate the positions where the linear B-scans are shown in panels (c) and (d). (c) B-scan extracted along the green-colored dashed line from the OCT volume at first visit. (d) B-scan extracted along the red-colored dashed line from the OCT volume. White arrows denote the mucoid effusion adjacent to the TM in panels (c) and (d).

Images from a patient with left ear chronic otitis media with effusion are shown in Fig. 6. An otoscopic evaluation revealed opacification of the TM potentially consistent with fluid in the middle ear with no apparent air-fluid levels, bubbles, or active otorrhea. OCT imaging demonstrated speckled signal intensity underlying a thickened TM distinct from the dark middle ear space evident in the OCT images of the right ear. The signal intensity drops off uniformly deeper into the middle ear space along the length of the TM, consistent with OCT imaging through a mucoid effusion with extensive scattering. A representative area with mucoid effusion adjacent to the TM is labeled by white arrows in Fig. 6. Following imaging, subsequent in-office myringotomy expressed thick yellow opaque mucoid fluid from the patients left ear, confirming the presence of fluid.

### 3.5 TM Perforation

**Fig. 7:**
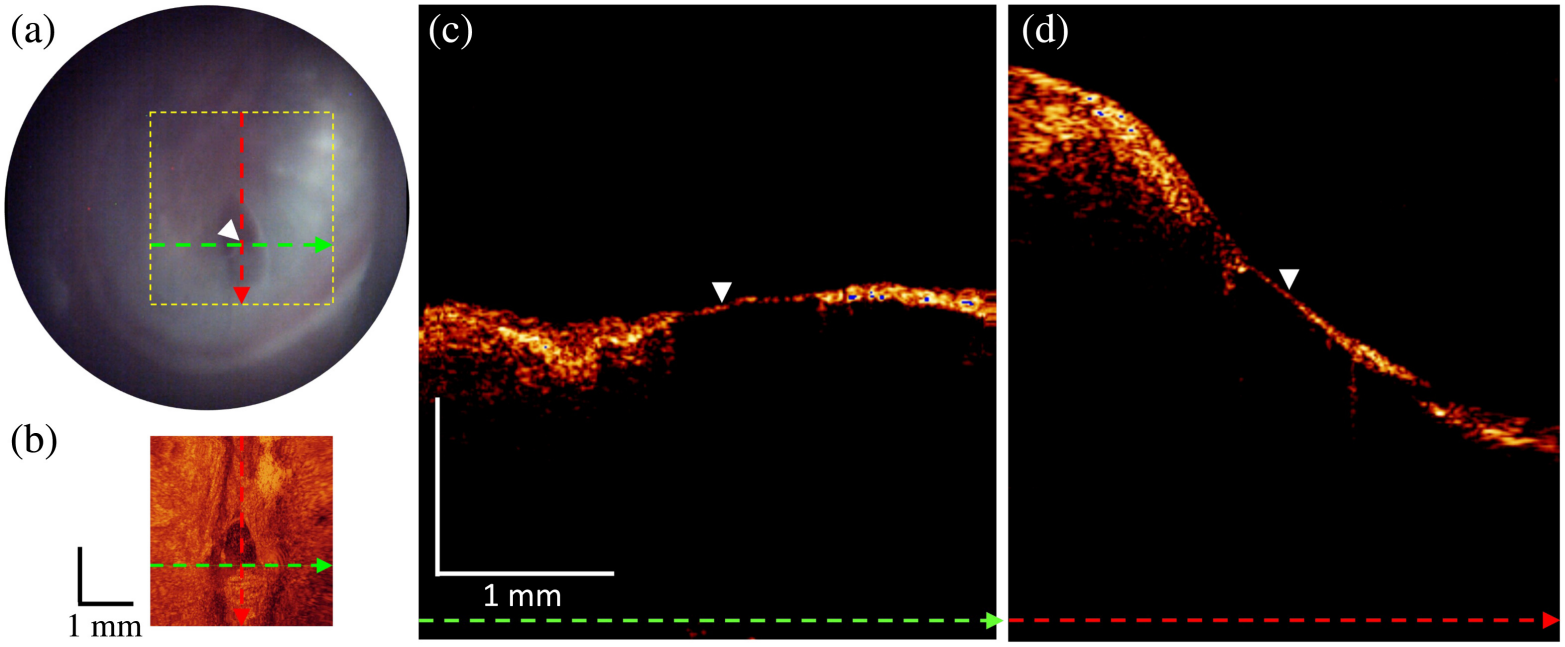
Otoscope and OCT image acquired from a healed thin TM. (a) A snapshot of the otoscopic video camera. Dashed colored lines indicate the positions where the linear B-scans are shown in panels (c) and (d). Dashed colored box indicates where the OCT volume was acquired. (b) *En face* image generated by SVP from an OCT volume inside of the dashed colored box on the otoscopic video image (a). Dashed colored lines indicate the positions where the linear B-scans are shown in panels (c) and (d). (c) B-scan extracted along the green-colored dashed line from the OCT volume. (d) B-scan extracted along the red-colored dashed line from the OCT volume. White arrows denote the healed TM in panels (c) and (d).

A patient with a history of chronic otitis media presented with a small elliptical TM perforation. On otoscopic examination during a 6-week follow-up exam, Fig. 7(a), the perforation appeared to be non-healing and patent into the middle ear cavity. However, the OCT cross-sectional images in Figs. 7(c) and 7(d) clearly show a thin layer of TM ∼68-μm thick, filling the space where the perforation had been. The OCT volume showed a healing elliptical perforation with minor and major axes of 300 and 400  μm, respectively, and a thin layer of tissue across the entire area.

### 3.6 TM Retraction

**Fig. 8:**
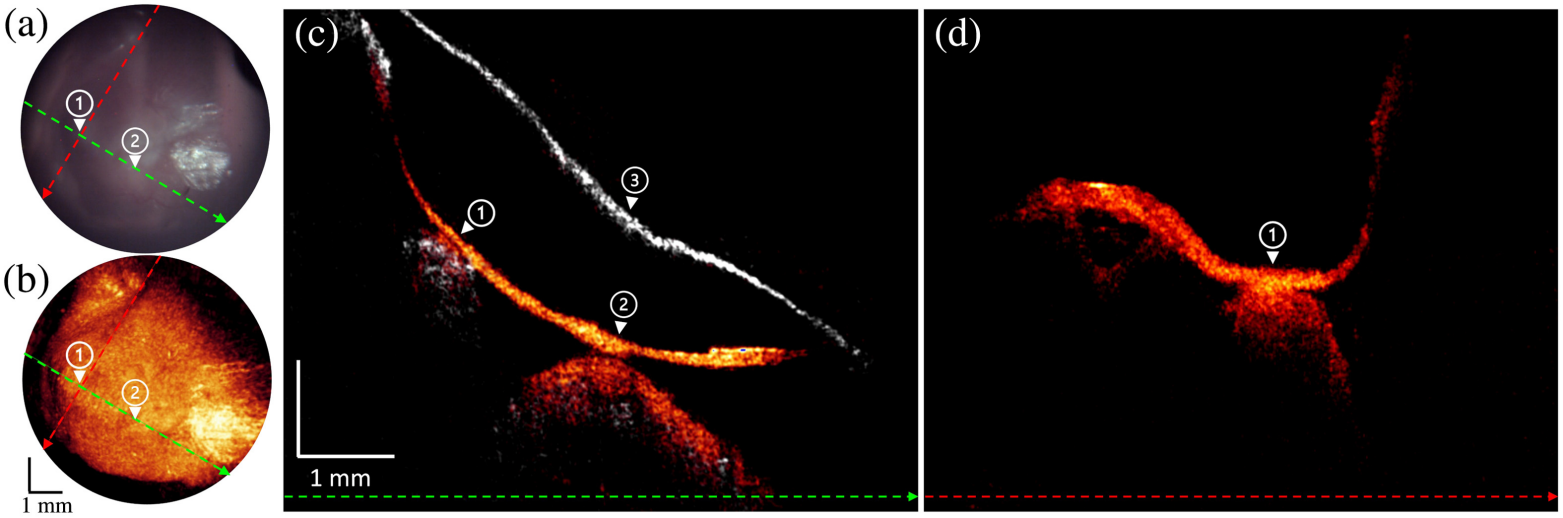
Otoscope and OCT image acquired from TM retraction patient. (a) A snapshot of the otoscopic video camera. Dashed colored lines indicate the positions where the B-scans are shown in panels (c) and (d). (b) *En face* image generated by SVP from an OCT volume. Dashed colored lines indicate the positions where the linear B-scans are shown in panels (c) and (d). (c) B-scan extracted along the green-colored dashed line from the OCT volume and overlaid with the gray-shaded OCT image (labeled 3) acquired from a normal condition. (d) B-scan extracted along the red-colored dashed line from the OCT volume. White arrows with circled numbers 1 and 2 denote that the TM touched the incus and was close to the cochlear promontory.

A patient with apparent TM retraction identified on otoscopic evaluation by an expert otologist was imaged. In the OCT cross-sections, Figs. 8(c) and 8(d), the TM is shown to be severely retracted. A co-registered normal TM is shown for reference in grayscale and denoted with a white arrow labeled 3. Inspection of the OCT volume showed that the TM was touching the incus and was very close but not touching the promontory. These are labeled in representative cross-sections by the circled numbers 1 and 2, respectively. TM contact with the incus brings an increased risk of bone necrosis and cholesteatoma formation.

### 3.7 Tympanic Membrane Protrusion with Air Pocket

**Fig. 9:**
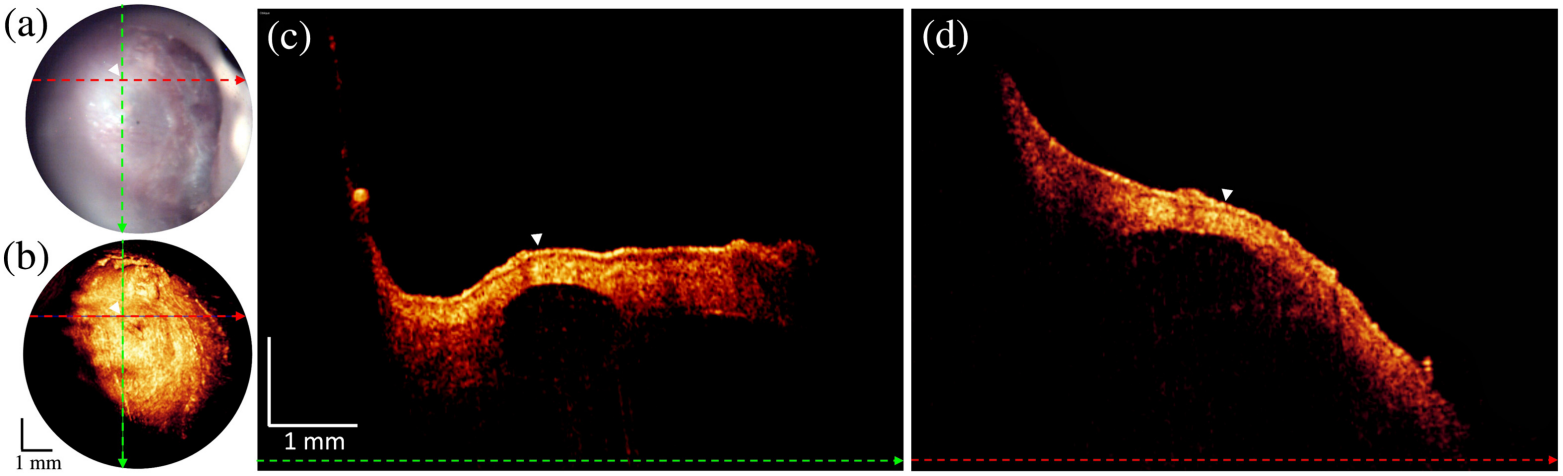
Otoscope and OCT image acquired from TM protrusion with an air pocket. (a) A snapshot of the otoscopic video camera. Dashed colored lines indicate the positions where the linear B-scans are shown in panels (c) and (d). (b) *En face* image generated by SVP from an OCT volume. Dashed colored lines indicate the positions where the linear B-scans are shown in panels (c) and (d). (c) B-scan extracted along the green-colored dashed line from the OCT volume. (d) B-scan extracted along the red-colored dashed line from the OCT volume. White arrows denote the TM protrusion with an air pocket.

A patient with a history of bilateral otosclerosis, stapedectomies (ear surgery to remove the stapes bone), and unknown pathology affecting the right ear TM presenting with decreased hearing and ear fullness was examined. They had seen several prior otologists with no reported clear diagnosis other than bulging of the TM or post-surgical changes. The otoscopic view appeared to show a partially white opaque TM possibly consistent with post-surgical scarring or middle ear mass of the TM posteriorly in Fig. 9(a). TM continuity with the superior-anterior quadrant of the ear canal was indeterminate with potential perforation on the otoscopic view. In Figs. 9(c) and 9(d), further OCT imaging demonstrated the presence of a ∼1.3-mm diameter air pocket within a body of thick scar tissue. The scar tissue, ∼420  μm thick, was continuous with the interior layer of the TM near the center, annotated with white arrows in Figs. 9(c) and 9(d), demonstrating no perforation on OCT imaging.

## 4 Discussion

Our new form factor and optical design for the OCT otoscope system provide a more compact hand-held component with a larger field of view than our prior work. The hand-held piece of the device is similar in size and shape to a standard battery-powered otoscope [see Fig. 1(f)]. The new optical design with a larger field of view, but otherwise similar optical performance as our prior work, comes at the cost of post-processing to recast the image, nominally collected in spherical polar coordinates, into Cartesian coordinates. Currently, we display the raw images in real time and do the resampling in postprocessing. The resampling could be done in real time; however, the quality of the raw images is good enough to easily see the relevant pathologies. The resampling is only important for making quantitative measurements, e.g., size of perforation, distance of TM to the incus or promontory, and TM thickness. Nevertheless, if real-time resampling proved necessary, it can be done using a GPU as demonstrated^25^ recently for a related resampling algorithm. In this system, we used a new custom laser source from Insight Photonics Solutions, Inc. (Lafayette, Colorado, United States) that uses a single laser chip. The single chip limits the available bandwidth but allows us to image at a line rate of 200 kHz and still maintain a large Nyquist depth of 17.7 mm. Although a large Nyquist depth is needed for middle ear imaging, 10 to 12 mm would probably be sufficient. A unique property of the Insight swept laser is the ability to change the sweep frequency. In the future, we can likely increase the line rate up to 300 kHz using this same laser; however, because the sampling rate remains fixed (400 MHz), our Nyquist depth would be reduced to 11.6 mm, which is still well within the range noted above.

The 2× increase in imaging speed over our prior work improves the image quality by reducing motion artifact, but it comes at the cost of axial resolution, which is reduced by approximately a factor of 2. Nevertheless, given the size of most anatomical features in the middle ear (e.g., TM thickness ∼100  μm and incus diameter ∼200  μm), the loss in resolution does not significantly degrade our ability to identify pathology within the TM and middle ear. Likewise, although we did not apply the algorithm here, we could improve our axia l resolution by up to a factor of 2 using a recently developed multi-window approach.^26^^–^^28^

Within the larger clinical study, for this report, we focused on examples in which the OCT imaging provides information that was not available from standard otoscopic examination and could be clinically important. Prior works with OCT devices have been used to characterize and aid in the diagnosis of a number of pathologies of the middle ear.^15^^–^^19^ An important example is otitis media with effusion, for which a non-scanning OCT device was developed into an FDA-approved diagnostic tool to help primary care physicians more accurately diagnose cases of otitis media with effusion.^29^ Our device and study build upon this growing body of work by not only being able to identify mucoid otitis media with effusion as shown in Fig. 6 within the volumetric image but also characterizing deeper structures and pathologies affecting the middle ear and TM.

Our first example was the growth of abnormal tissue on the TM thought to be due to active myringitis, inflammation of the outer squamous layer of the TM. Normally, this pathology is diagnosed through otoscopic examination that demonstrates signs of inflammation with ulceration, yellow crusting, or polypoid growths.^30^ Treatment often involves curettage of the abnormal tissue and antibiotic drops.^31^^,^^32^ In the case that we describe in Fig. 5, the abnormal buildup of scar-like tissue and the lack of classical signs of myringitis made diagnosis difficult from just visualization with a standard otoscope. The OCT volumetric images showed that the abnormal tissue was attached to the TM and not just laying on top of the TM. Removing the tissue by debridement could have resulted in tearing of the TM, producing a perforation. Instead, ear drops were administered over a 6-week period.

TM perforations are typically able to be resolved on otoscopic evaluations. They are important because long standing non-healing perforations have been shown to be associated with increased risk of middle ear infections and cholesteatomas with the middle ear space open to the external environment.^33^ What are harder to determine are the intermediate stages of perforation healing and smaller perforations in which otoscopic evaluation are again subject to a health professional’s subjective interpretation. In the case that we describe, the prior perforation appeared to be patent using a standard otoscopic view, but OCT imaging showed a healing layer of TM was present, deferring need for further intervention. This illustrates the potential for OCT imaging to be used for monitoring and managing perforations.

Retraction pockets have not been previously characterized by OCT. In the patient described above, the OCT images clearly showed what areas of the TM were retracted, the relative depth of retraction, and if it was contacting key structures in the middle ear. Current methods of grading the severity of retraction use contact with important structures as a measure and include the use of the Tos and Poulson or Sade scale.^34^ However, grading is based on subjective interpretations by a physician through 2D otoscopic evaluation and hence is not used often due to the difficulty in accurately applying the scales only using traditional 2D information and middle ear pressures.^34^ With OCT, healthcare professionals would be able to apply these scales using more accurate and objective 3D information.

Finally, we describe a case in which a patient appeared to have a white mass in the middle ear cavity via otoscopic view, but OCT revealed the presence of a scar air pocket that was causing the TM to bulge outward. This demonstrated how the device can be used to characterize patterns of scarring and how it can disrupt the normal architecture of the TM and thus affect the conduction of sound and hearing. Future applications could involve using this ability of OCT to distinguish between the presence of masses versus structural changes such as that described here to determine the need for a further medical workup or investigation.

## 5 Conclusion

The operation of the OCT otoscope device described above is sufficiently easy, so a single person can collect a set of images from a patient in seconds to minutes. This has allowed us to regularly image in the USC hearing clinic without disrupting the workflow within the clinic. The improved imaging speed and field of view have aided in the collection of images that provide information not readily available from a traditional otoscope examination. These OCT findings and the pathological information presented in this study serve to further demonstrate hand-held OCT’s ability to be a useful clinical tool.

## Supplementary Material





## Data Availability

Imaging data and related processing code available upon reasonable request from the corresponding author.
